# Conjunctival melanocytic naevus: Diagnostic value of anterior segment optical coherence tomography and ultrasound biomicroscopy

**DOI:** 10.1371/journal.pone.0192908

**Published:** 2018-02-14

**Authors:** Eszter Vizvári, Ákos Skribek, Nóra Polgár, András Vörös, Pál Sziklai, Edit Tóth-Molnár

**Affiliations:** 1 University of Szeged, Department of Ophthalmology, Szeged, Hungary; 2 “Szemambulancia” Ophthalmology Private Practice, Szeged, Hungary; 3 University of Szeged, Department of Pathology, Szeged, Hungary; University of Debrecen, Faculty of Medicine, HUNGARY

## Abstract

**Purpose:**

Conjunctival naevi are the most frequently diagnosed primary melanocytic lesions of the conjunctiva. The clinical manifestations are greatly variable which may result in diagnostic difficulties and differential diagnostic confusions. Therefore aims of the present study were: 1) to assess the morphologic features of conjunctival naevi; 2) to delineate the anterior segment optical coherence tomography (AS-OCT) characteristics of these lesions; 3) to compare AS-OCT and ultrasound biomicroscopy (UBM) as diagnostic tools in these alterations and 4) to correlate histological results with the AS-OCT pictures in case of surgically excised naevi.

**Methods:**

All lesions were photo-documented. AS-OCT and UBM (over the age of 18 years) were performed. Surgically excised lesions were admitted to histological examinations.

**Results:**

In our series of 57 conjunctival naevi, 54.4% were highly pigmented, 15.8% proved to be amelanotic. AS-OCT could detect intralesional cysts in 61.4% of the naevi, while slit-lamp and UBM proved to be less sensitive (40.3% vs. 28.5%). UBM could visualize the posterior margins of all naevi, while AS-OCT proved to be less sensitive with the detection of 89.4% of posterior naevus margins. Thickness of the conjunctival epithelial layer could be measured with AS-OCT in case of subepithelial naevi, while no distinct epithelial layer could be detected in compound and junctional naevi.

**Conclusions:**

Superiority of AS-OCT over UBM was demonstrated in visualizing internal structures of conjunctival naevi. UBM proved to be a better tool in highly pigmented and remarkably elevated naevi. Correlation was found between the histological type of the naevus and the thickness of the epithelial layer covering the lesion.

## Introduction

Conjunctival naevi are the most frequently diagnosed benign melanocytic lesions of the conjunctiva. Clinical manifestations of these alterations are greatly variable which may result in diagnostic difficulties and differential diagnostic confusions. Although the potential of conjunctival naevi for malignant transformation is low (less, than 1%), but up to 20% of conjunctival melanomas can arise from pre-existing naevi [1]. In contrast to benign naevi, malignant melanoma of the conjunctiva is a rare entity but the incidence of this malignancy has been continuously increasing worldwide [2]. Considering the high potential of conjunctival melanoma for developing metastatic disease, early detection or even primary prevention of the tumour development is of great importance [3, 4, 5]. These efforts include mainly the recognition and adequate follow-up of the potentially precursor lesions such as conjunctival naevus.

Until advances in non-invasive *in vivo* imaging techniques in the past two decades, clinical diagnosis and follow-up of conjunctival naevi was based mainly on slit-lamp examinations and photo-documentations. In 1990, high frequency ultrasound biomicroscopy (UBM) was introduced to visualize the anterior portion of the eye with greater details than earlier [6]. This diagnostic modality enables the examination of the anterior segment of the eye in depth with relatively high resolution. In the 50 MHz mode, the resolution can be approximately 25 micrometer and the depth of tissue penetration is 5–6 mm. Among the new diagnostic modalities, continuously evolving techniques of optical coherence tomography (OCT) is a non-contact tool for higher resolution providing cross-sectional *in vivo* imaging of ocular tissues. OCT uses long wavelength light instead of ultrasound to capture images, thus light penetration is the limitation of this technique as opaque tissues block light transmission [7]. OCT has revolutionized ocular imaging on numerous fields of application. Novel modifications of OCT have allowed visualization of the anterior segment of the eye (anterior segment OCT: AS-OCT), and opened a new horizon not only in the detailed visualization of the cornea and the angle structures, but also in the diagnosis of conjunctival pathology [8]. Morphological characteristics of different pathological lesions can be examined beneath the ocular surface plane with axial resolution of 4–6 μm and vertical resolution of less, than 20 μm using the spectral domain signal analysis mode. The technical development is continuous and fast, enabling more detailed visualization of anterior segment pathologies (ultrahigh speed swept source AS-OCT) [9].

Aims of the present study were 1) to assess the morphological features of conjunctival naevi in a South Hungarian population; 2) to delineate the AS-OCT characteristics of conjunctival naevi; 3) to compare AS-OCT and ultrasound biomicroscopy (UBM) as diagnostic tools in these alterations; 4) to correlate the OCT characteristics of the lesions with the histopathological examination results in surgically removed conjunctival naevi.

## Materials and methods

The study was approved by the Institutional Review Board of the University of Szeged, and was conducted in accordance with the principles of the Declaration of Helsinki. Patients gave their informed consent prior to OCT and UBM examinations and prior to surgical interventions.

### Patients

Patients with conjunctival naevi examined at the Department of Ophthalmology, University of Szeged between September 2013 and September 2015 were included in the present study. Upon inclusion, the diagnosis was based on the clinico-mophological appearance of the lesion. Collected demographic data included age and gender. All patients were examined with AS-OCT. UBM examination was performed only after the age of 18 years as it requires eyebath to obtain images (eyebath can be inconvenient in younger patients). Surgical excision was performed for multiple reasons: 1) in suspected malignancies, 2) in the case of suspicious changes, or 3) for cosmetic reasons (disturbing, but clinically benign lesions).

### Clinical evaluation

All conjunctival melanocytic lesions were photo-documented. The following clinical features were recorded: location of the tumour (bulbar, caruncular, plical, tarsal-intermarginal); degree of pigmentation (dark, moderately-lightly pigmented, amelanotic), presence of intrinsic cysts visible with slit lamp examinations, presence of feeding vessels. Besides the documentation of conjunctival alterations, iris colour was recorded and assessed on a 3-grade scale: 1: darkly pigmented (dark brown); 2: intermediately pigmented (light brown-hazel); 3: lightly-fairly pigmented (green-gray-blue). The presence of iris freckles and iris naevi was also documented.

### AS-OCT examination

All OCT examinations were performed by the same well-trained ophthalmologist (EV) using TOPCON 3D OCT-2000 (Topcon Corporation, Tokyo, Japan) spectral domain OCT equipment (operating at 840 nm wavelength). OCT images were captured using the 3D scan mode and were analysed according to the considerations similar to those described by Shields et al.: configuration (flat, dome); intrinsic optical characteristics of the lesions (solid or hollow); internal tumour pattern (homogeneous or heterogeneous); presence of intrinsic cysts; presence of posterior shadowing (mild-moderate-severe); naevus thickness and basal diameter [10]. As dimensions of the naevi vary across images, the largest parameters in all three dimensions were measured (largest horizontal and vertical dimensions and largest elevation). Thickness of the conjunctival epithelial layer above the anterior surface of the naevus was measured on the OCT image in surgically removed naevi. Attempt was made to correlate the thickness of the epithelial layer with the histological type of the naevus (junctional, compound, subepithelial).

### UBM examination

All UBM examinations were performed by the same well-trained ophthalmologist (AS) using Sonomed VuMax UMB 50 MHz equipment (Sonomed Escalon, Berlin, Germany). Subjects were examined in the supine position with the eye immersed in saline solution using an eyecup. Images captured with OCT and UBM examinations were compared using the following considerations: quality of tumour margin visualization (anterior-posterior); visibility of internal tumour structures (presence of intralesional cysts); presence of posterior shadowing.

### Histological examinations

Surgically excised lesions were admitted to histological examinations. Haematoxylin-eosin staining was performed in the case of all excised lesions. Immunohistochemical detection of S100 / melan A / HMB45 were performed.

### Statistical analysis

SPSS software version 17.0 (SPSS, Chicago, IL, USA) was used for statistical analysis.

## Results

### AS-OCT, UBM and conjunctival naevi

**Fig 1:**
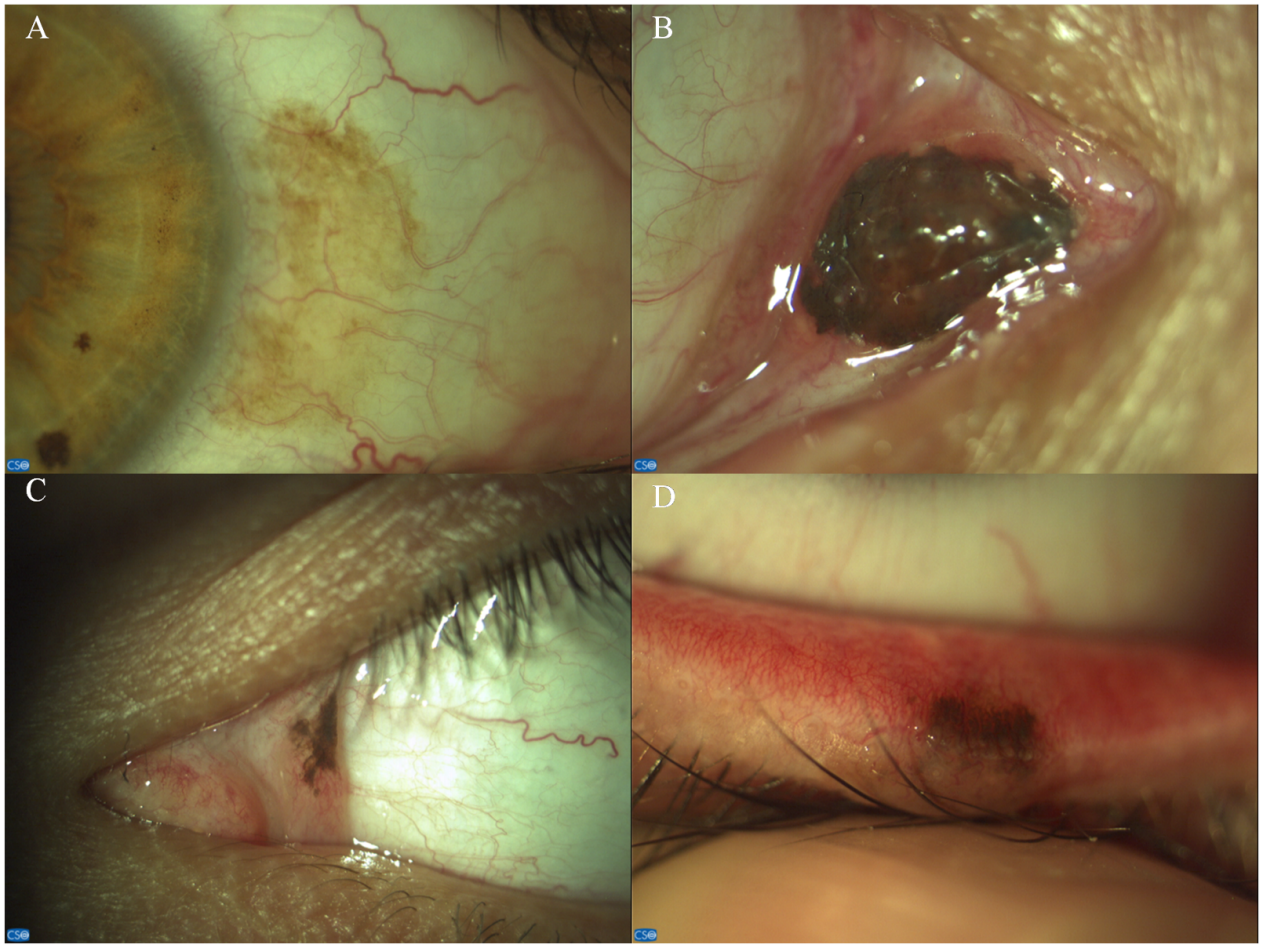
Localization of conjunctival naevi: Bulbar (A), caruncular (B), plical (C), intermarginal (D).

**Fig 2:**
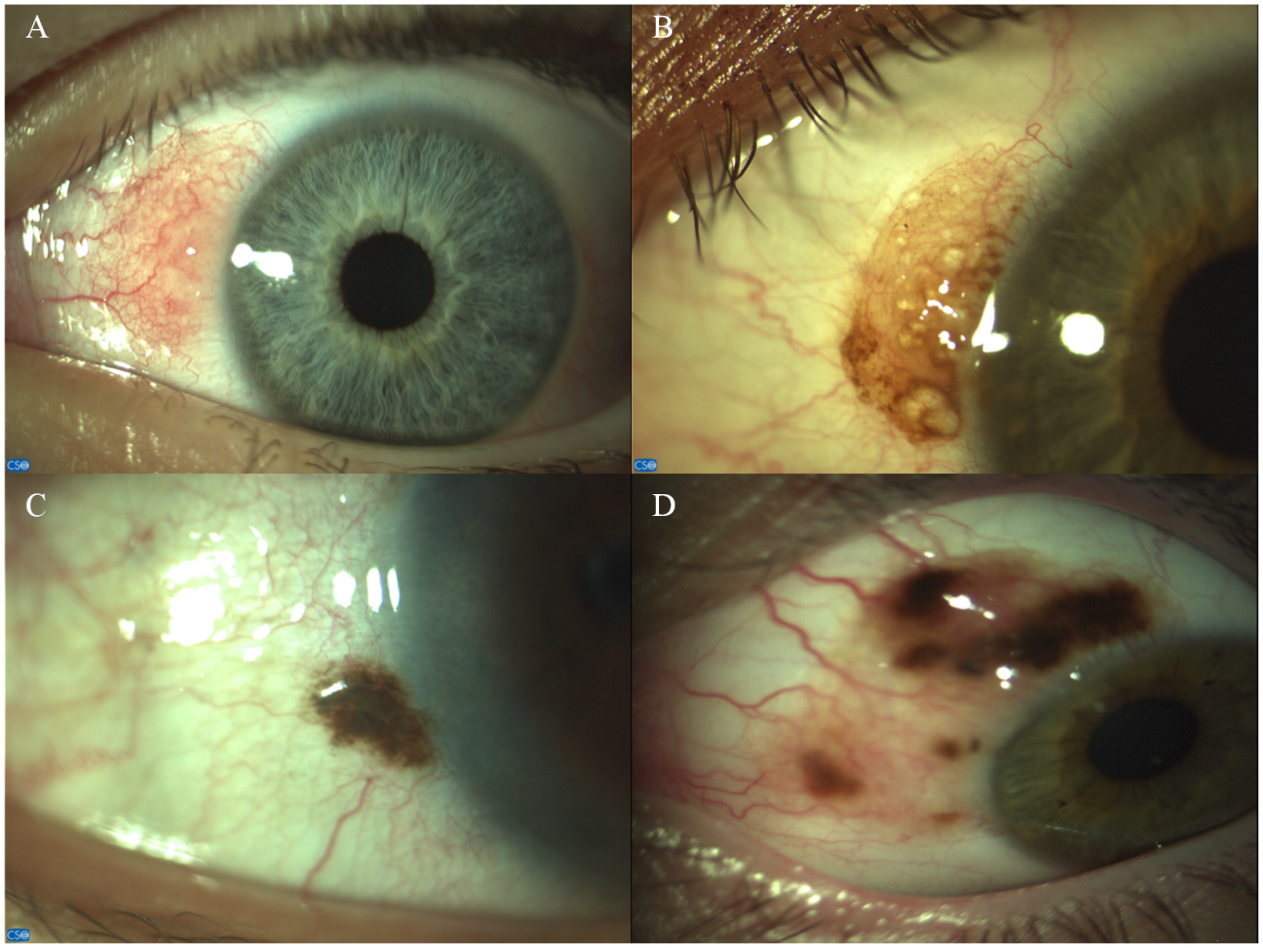
Clinical appearances of conjunctival naevi are extremely variable. Anterior segment pictures of amelanotic and cystic (A), moderately pigmented and cystic (B), small, strongly pigmented (C) and giant juvenile (D) conjunctival naevi.

**Fig 3:**
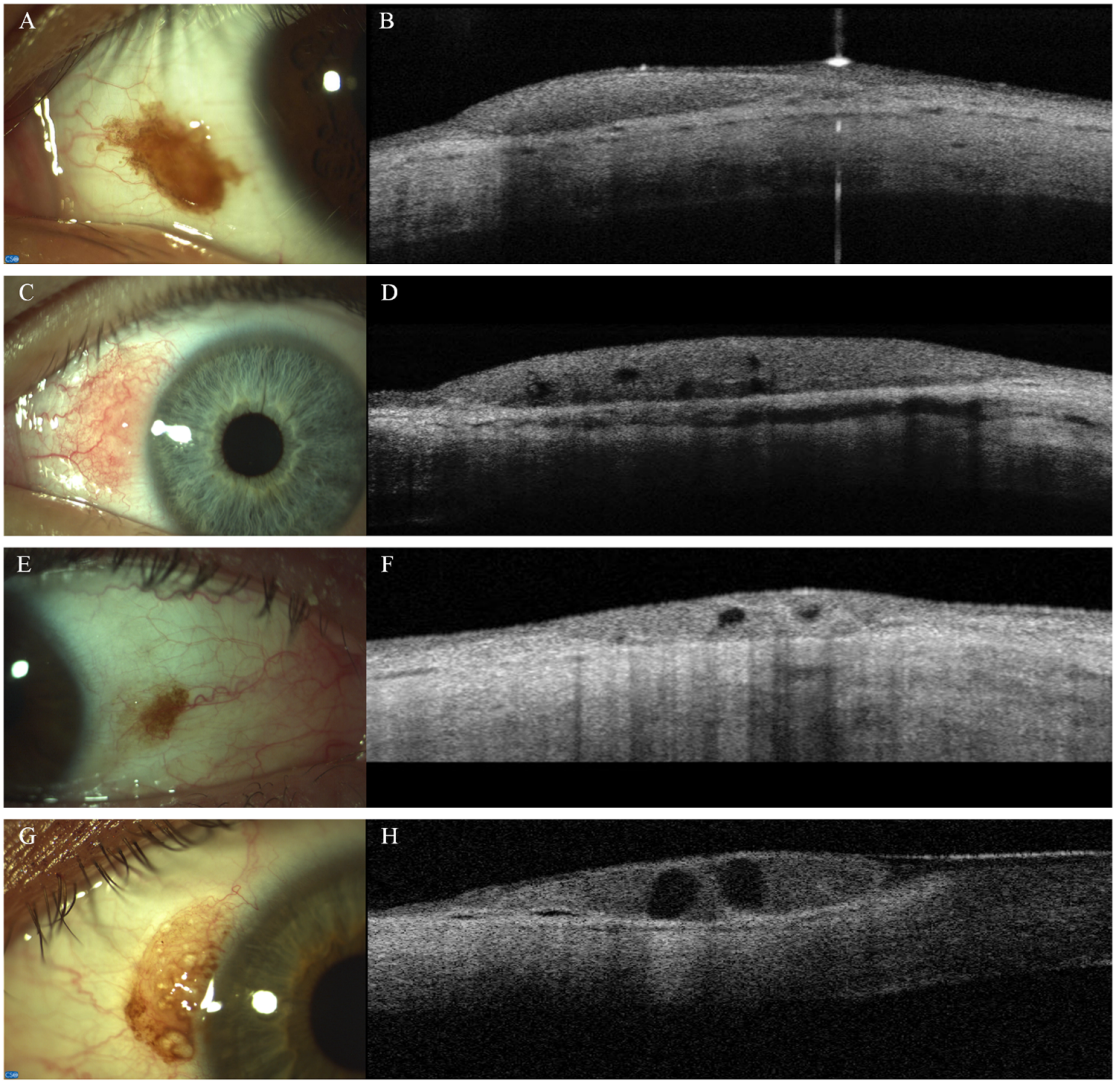
Clinico-morphological features (A, C, E, G) and anterior segment optical coherence tomography (AS-OCT; B, D, F, H) of conjunctival naevi. Pigmented lesion (A) without cystic alterations on AS-OCT (B). Amelanotic naevus with macroscopically visible cysts (C), which are confirmed with AS-OCT examination (D). Small pigmented conjunctival naevus: cystic alterations cannot be visible on slit-lamp examination (E), intralesional cysts can be detected only with AS-OCT (F). Pigmented conjunctival naevus: intralesional cysts can be visualized both with slit-lamp examination (G) and with AS-OCT (H).

**Fig 4:**
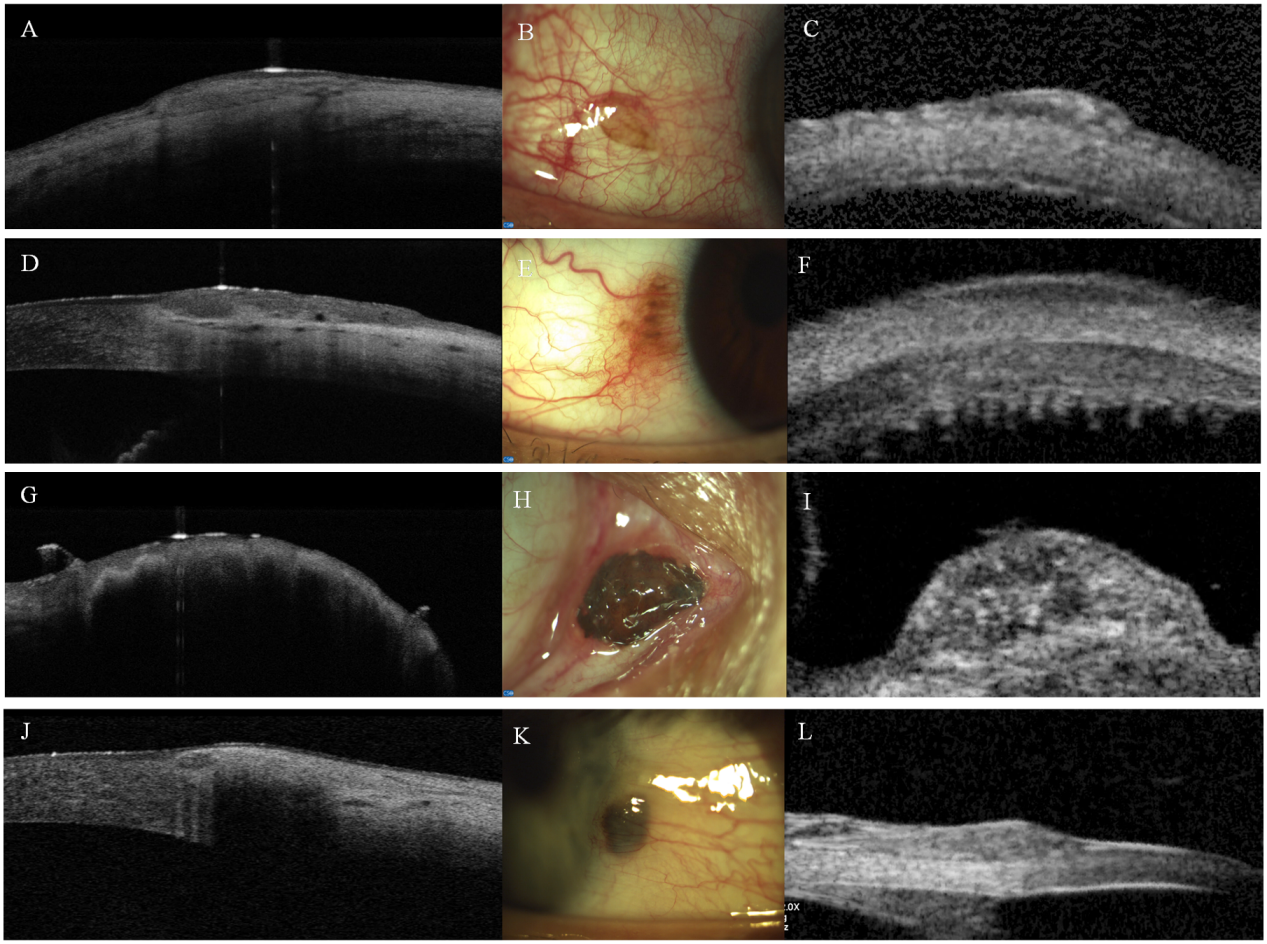
Comparison of anterior segment optical coherence tomography (AS-OCT, left column: A, D, G, J) and ultrasound biomicroscopy (UBM, right column: C, F, I, L) for imaging conjunctival naevi. Middle column: clinical eppearane of the naevi (B, E, H, K). Intralesional morphology of pigmented bulbar naevi (B, E) can be depicted on AS-OCT examinations in more details (A, D) compared to UBM examinations (C, F). In contrast, highly pigmented caruncular (H) and bulbar naevi (K) can be completely visualized only with UBM examinations (I, L), as severe shadowing limits the usefulness of AS-OCT (G, J).

**Fig 5:**
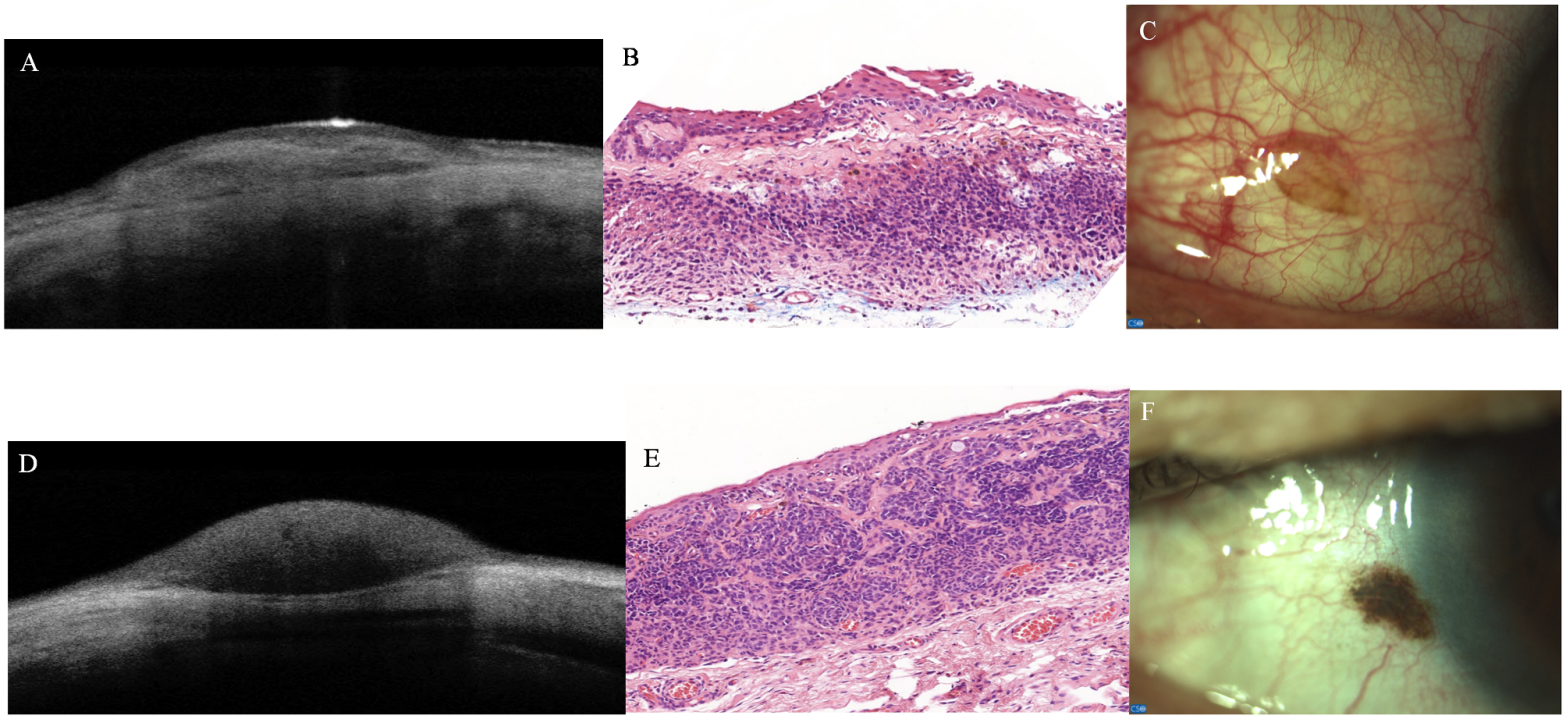
Subepithelial conjunctival naevus: anterior segment optical coherence tomography (AS-OCT) (A); histopathological appearance (B, hematoxylin-eosin, x10) and clinical appearance (C). Junctional conjunctival naevus: AS-OCT feature (D); result of histopathological examination (E, hematoxylin-eosin, x10); clinico-morphological appearance (F).

Fifty-six eyes of 56 patients with 57 conjunctival naevi were examined (25 female [44.6%], mean patient age: 32.5 years, range 4–71 years, all patients were Caucasian), one patient exhibited 2 conjunctival naevi on the same eye. Clinical characteristics of the naevi are summarized in Table 1 (Figs 1 and 2). One giant juvenile conjunctival naevus was excluded from the morphometric analysis (naevus thickness, horizontal and vertical diameter) harbouring approximately 40 percent of the bulbar conjunctiva (Fig 2D). Most of the naevi were darkly pigmented (54.4%) located on the bulbar conjunctiva (73.7%), 15.8% of the naevi proved to be amelanotic. Mean age of patients was considerably lower in the amelanotic naevi group compared to the group of patients with pigmented naevi (amelanotic: mean 9 years [range 4–13] vs. melanotic: mean 37.2 years [range 13–71]. Intrinsic cysts were detected in 40.3% of the naevi with slit-lamp examination, while 61.4% of the naevi had feeder vessels. All naevi were examined with AS-OCT, results are given in Table 2 (Fig 3). With the use of AS-OCT, all lesions were homogeneously solid. Intrinsic cysts were detected in 61.4% of the naevi. Good resolution was found with the naevus margins (anterior: 100%, posterior: 89.4%, lateral: 96.5%). Some degree of optical shadowing posterior to the naevus was found in 66.7% of the lesions. UBM examination was performed on 21 eyes of 21 patients with 21 conjunctival naevi. Posterior margins of the naevi could be visualized in all lesions, intrinsic cysts could be detected in 28.5% of the cases. OCT and UBM characteristics of the 21 naevi examined with both methods are summarized in Table 3 (Fig 4). UBM examination proved to be superior in the visualization of the posterior margin (100% vs. 85.7%), while OCT was more precise in the detection of intralesional cysts (57.1% vs. 28.5%). Twenty-four naevi were excised and admitted to histopathological examination. The clinically established diagnosis of conjunctival naevi were confirmed histologically in all cases (compound naevus: 12 [50%], junctional naevus: 4 [16.7%], subepithelial naevus: 8 [33.3%]). In the case of excised lesions, preoperative OCT characteristics of the conjunctival epithelial layer (visibility, thickness) and the results of the histological examinations are given in Table 4. Conjunctival epithelial layer could be visualized with preoperative AS-OCT only in the subepithelial forms while no distinct conjunctival epithelium could be detected in the junctional and compound types (Fig 5).

**Table 1:** Clinical features of conjunctival naevi (total number of naevi = 57).

Clinical characteristics of naevi	Number (%)
**Pigment status**	
dark	31 (54.4)
moderate –light	17 (29.8)
amelanotic	9 (15.8)
**Location**	
bulbar	42 (73.7)
caruncular	8 (14.0)
plical	5 (8.8)
tarsal-intermarginal	2 (3.5)
**Naevus configuration**	
flat	24 (42.1)
dome	33 (57.9)
**Associated features**	
feeder vessels	35 (61.4)
intrinsic cysts	23 (40.3)

**Table 2:** Anterior segment optical coherence tomography (AS-OCT) features of conjunctival naevi (total number of naevi = 57).

AS-OCT feature	Number (%)
**Optical characteristics**	
solid	57 (100)
hollow	0 (0)
**Internal pattern**	
homogeneous	57 (100)
heterogeneous	0 (0)
**Naevus thickness** (mean [range] in mm)	0.43 (0.13–0.81)^†^
**Naevus vertical diameter** (mean [range], in mm)	3.12 (1.16–6.72)^†^
**Naevus horizontal diameter** (mean [range], in mm)	3.13 (1.21–4.94)^†^
**Visibility of naevus margins**	
anterior	57 (100)
posterior	51 (89.4)
lateral	55 (96.5)
**Intrinsic cysts**	35 (61.4)
**Posterior tumour shadowing**	
non	19 (33.3)
mild-moderate	29 (50.9)
severe	9 (15.8)

^†^n = 56 (one giant juvenile conjunctival naevus was excluded from the morphometric analysis)

**Table 3:** Comparison of anterior segment optical coherence tomography (AS-OCT), ultrasound biomicroscopy (UBM) and slit-lamp (SL) examinations in naevus margin visualization, in the detection of intralesional cysts and in posterior naevus shadowing in 21 conjunctival naevi (21 eyes).

	UBM number (%)	OCT number (%)	SL number (%)
**Visualization of posterior tumour margin**	21 (100)	18 (85.7)	-
**Detection of intrinsic cysts**	6 (28.5)	12 (57.1)	8 (38.1)
**Posterior naevus shadowing** (mild-moderate and severe)	0 (0)	12 (57.1)	-

**Table 4:** Results of histological examination of the excised naevi (n = 24). Epithelial layer thickness above the naevus was measured on anterior segment optical coherence tomography images before surgical excision.

Histological type of the naevus	Number (%)	Thickness: mean [range] (in μm)/ visibility of epithelial layer
Junctional	4 (16.7)	not visible
Compound	12 (50.0)	not visible
Subepithelial	8 (33.3)	79.2 [76–90]

### Conjunctival melanocytic lesions, eye colour and pigmented alterations of the iris

Eye colour, the presence of iris freckles and iris naevi among patients with conjunctival melanocytic lesions are summarized in Table 5. More, than 40% of the conjunctival naevi patients had darkly pigmented irides, while 17.8% of the patients had light iris colour (green-gray-blue). Iris naevus could be observed in 26.8% of the patients.

**Table 5:** Conjunctival naevi, eye colour, and the presence of iris freckles and naevi. Total number of eyes with naevus = 57.

	Eyes with conjunctival naevus number (%)
**Iris colour**	
dark brown	23 (41.1)
light brown-hazel	23 (41.1)
green-gray-blue	10 (17.8)
**Number of eyes with**	
iris freckles only	16 (28.6)
iris naevus only	6 (10.7)
both iris freckles and naevus	9 (16.1)
**Sum of eyes with iris naevus**	15 (26.8)
**Sum of eyes with pigmented iris alterations**	31 (55.3)

All relevant data can be found in the Supporting Information file (S1 Table).

## Discussion

During the past few years new evolving diagnostic modalities have become more widely available in the clinical practice to improve the diagnostic accuracy of ocular surface alterations. Zhang et al. described in vivo, cross-sectional, full thickness AS-OCT morphology of the healthy human conjunctiva [11]. Histologically, the bulbar conjunctiva consists of epithelial and stromal layers. As epithelial layer is a well-arranged structure with low light scatter, the reflectivity of the epithelium is low. In contrast, the conjunctival stroma highly scatters incident light which results in hyper-reflectivity of this layer.

There are limited reports about OCT examinations of conjunctival tumours. Shields et al. analysed AS-OCT images of 22 conjunctival naevi, and concluded that this method can be helpful in visualizing tumour boundaries and cysts within the tumour [10]. Another recent report described AS-OCT observations of thickened and hyper-reflective epithelium in squamous conjunctival neoplasia [9]. In a recently published article Shousa and colleges correlated OCT examination results with histopathological findings of 54 eyes possessing various conjunctival pathological alterations among them 2 cases of conjunctival naevus were described [12].

There are only few published reports comparing AS-OCT and UBM images of anterior segment tumours and even less in relation with conjunctival tumours. Bianciotto et al. assessed 200 cases of anterior segment tumours, but only 6 cases of conjunctival lesions were included [13]. Pavlin and colleges reported UBM and OCT characteristics of 18 eyes with iris and ciliary body tumours, but no eye with conjunctival pathology was presented [14]. Buchwald and colleges studied 13 conjunctival lesions with AS-OCT and UBM, 4 of them were conjunctival naevi [15].

To the best of our knowledge, the present series of 57 conjunctival naevi examined with OCT, 21 out of them also with UBM is the largest series of conjunctival naevi analyzed.

Only few articles have focused on the clinical features of conjunctival naevi [16]. The largest series with the detailed description of the clinical characteristics of 410 conjunctival naevi was published by Shields et al [17]. In our series of 57 conjunctival naevi, approximately 60% of the lesions exhibited highly pigmented, dome shaped morphology, 61.4% had feeder vessels and 15.8% proved to be amelanotic. We detected one case of giant conjunctival naevus (Fig 2D) which is a rare entity representing approximately 5% of conjunctival naevi according to the only available survey of 32 cases [18]. Considering the clinico-morphological appearance (large size and variegate pigmentation), these lesions can cause differential-diagnostic difficulties as they can be confused with malignant melanoma. Giant nevi represent a rare sub-group of melanocytic conjunctival alterations. Considering the extended conjunctival involvement and variegated clinic-morphological appearance. In case of amelanotic naevi, AS-OCT can provide helpful additional information to improve diagnostic accuracy. Frequent misdiagnosis of amelanotic naevi can lead to unnecessary anti-inflammatory treatment (scleritis, episcleritis, pinqueculitis are the most important differential-diagnostic pathologies). Mean patients age was considerably lower in the amelanotic group compared to the melanotic (9 years vs. 37.2 years) which can provide helpful information regarding the maturation process of the naevi. AS-OCT proved to be a very precise tool in determining morphometric parameters of conjunctival naevi, including basal dimensions and thickness, internal features and intralesional cysts. Intralesional cysts were detected in 61.4% of the naevi with OCT, less with slit lamp examinations (40,3%) and UBM proved to be the less sensitive tool from this aspect (28.5%). Smaller cysts can remain unobserved with slit lamp examinations, while OCT can detect deeply localized cysts within the naevi. As epithelial cell-walled intralesional cysts are the “memory” of epithelial origin in conjunctival naevi, they can be considered as signs of chronicity suggesting benign nature of the lesion but without disclosing malignancy. Clinical features of conjunctival melanoma and naevi can overlap: feeder vessels can be detected both in conjunctival melanoma and conjunctival naevi. Therefore detection of intralesional cysts can be one of the key point in differentiating conjunctival melanoma from naevi. Posterior margin could not be detected with AS-OCT in 6 out of 57 naevi due to strong posterior shadowing of highly pigmented, dome shaped caruncular (3 cases) and bulbar (3 cases) naevi. These findings reflect the limitations of AS-OCT examinations in case of conjunctival naevi: dark pigmentation (posterior shadowing), and also the thickness and the location (caruncula) of the naevi may limit the visualization of conjunctival naevi with OCT. In contrast, UBM examinations could visualize posterior margins of the lesions in all cases. In summary, UBM proved to be a better tool in the visualization of the posterior margins of darkly pigmented naevi. We have correlated the AS-OCT characteristics and the histological type of the naevi. AS-OCT images could demonstrate the involvement of the epithelial layer in compound and junctional naevi since no distinct epithelial layer could be observed in these alterations. In contrast, epithelial layer of the conjunctiva could be visualized and measured in subepithelial naevi. This latter finding can help to investigate the natural course of naevus evolution and may help to understand the process underlying malignant transformation of the lesions.

We assessed the iris colour and the presence of iris freckles and naevi among patients with conjunctival melanocytic lesions. Percentage distribution of iris colour among patients with conjunctival naevi were similar to the eye colour pattern of the general Caucasian population. We could not find literature data regarding the prevalence of pigmented iris alterations in a relatively large population of patients with conjunctival melanocytic lesions. Twenty-seven percent of conjunctival naevi patients had at least one iris naevus. There are accumulating evidences, that the presence of iris naevus can be considered as a risk indicator for the development of uveal melanoma [19]. Iris naevus has been reported to occur in 4–6% of the Caucasian population. In accordance with the literature data, the prevalence of iris naevus proved to be 5.6%-6.2% in our earlier investigations [20, 21, 22]. The occurrence of iris naevus in our present study population was substantially higher. Further studies are needed to investigate the ocular melanoma risk indicator role of iris naevus in relation to the coincident presence of conjunctival melanocytic lesions.

## Conclusions

In summary, our present study demonstrated the superiority of AS-OCT over UBM examinations in visualizing internal structures of conjunctival naevi in fine details. Epithelial and stromal layers of the conjunctiva around the naevi could also be depicted with AS-OCT. Correlation was found between the histological type of the naevus and the thickness of the epithelial layer covering the lesion therefore AS-OCT examination results may predict the histological subtype of the naevi.

UBM examination proved to be a better tool in case of highly pigmented and remarkably elevated naevi. The phenotypic marker role of the high prevalence of iris naevi among patients with melanocytic conjunctival lesions should be in the scope of further investigations.

## Supporting information

S1 Table All data from the conjunctival naevi examined can be found. (XLSX)
